# Navigating Severe Obsessive-Compulsive Disorder (OCD) With Food Obsessions: A Case Report on Diagnostic Complexity and Atypical Presentation

**DOI:** 10.7759/cureus.89776

**Published:** 2025-08-11

**Authors:** Lubna Lutfi, Basma S Hussein, Mazin A Mukhtar

**Affiliations:** 1 Psychiatry, Al Amal Psychiatric Hospital, Emirates Health Services, Dubai, ARE; 2 Psychiatry, Maudsley Health, Dubai, ARE

**Keywords:** atypical antipsychotic, compulsions, obsessions, ocd/anxiety disorders, refeeding, selective serotonin reuptake inhibitor (ssri)

## Abstract

This case report discusses a 29-year-old male with severe obsessive-compulsive disorder (OCD) who presented with food-related obsessions and compulsions, leading to extreme malnutrition, reflected in a dangerously low body mass index of 13.7 kg/m². His obsessions revolved around keeping his digestive tract completely empty to avoid illness. To achieve this, he restricted his food intake, spent long hours in the toilet, and engaged in excessive washing. These behaviors resulted in significant physical deterioration that eventually required emergency medical intervention. Unfortunately, the initiation of treatment led to refeeding syndrome, which further complicated the case. Due to his unique symptoms and acute presentation, this case highlights the diagnostic and therapeutic challenges posed by atypical presentations of OCD. It reinforces the importance of a multidisciplinary team involving medical, psychiatric, and nutritional departments working collaboratively. Close monitoring for complications, such as refeeding syndrome, is essential. Psychoeducation for both patients and families remains crucial to support adherence to the treatment plan and improve outcomes.

## Introduction

Obsessive-compulsive disorder (OCD) is a mental health condition that is characterized by the presence of “obsessions” or unwanted, intrusive, and repetitive thoughts. These thoughts lead to immense anxiety and stress for the individual experiencing them. In many cases, individuals with obsessive thoughts develop certain routines or acts, termed “compulsion”, to relieve the building stress [1].

The etiology of OCD is highlighted in a few hypotheses: hyperactivity in the cortico-striatal-thalamic loop circuits (CSTS), which contributes to the rigidity in thinking, impairment in serotonin and dopamine systems, which is evident by the relief of symptoms with the use of serotonergic or dopaminergic pharmacological agents, and finally the presence of high levels of glutamate, an excitatory neurotransmitter, in the brain in people suffering with OCD [2].

A summary of the OCD diagnostic criteria provided in the Diagnostic and Statistical Manual of Mental Disorders, Fifth Edition, Text Revision (DSM-5-TR) includes the presence of obsessions, compulsions, or both, that must be time-consuming (over one hour per day) or cause significant impairment in socio-occupational functioning, and cannot be attributed to substances or other medical or mental health conditions. It is further divided into specifiers, most commonly conditions with good or fair insight, then conditions with poor insight, and rarely, conditions with absent insight/delusional beliefs [3]. 

Despite the general understanding of the condition, bizarre and atypical presentations of OCD are possible and have been reported in the literature [4,5]. Our case presented an unusually acute and life-threatening clinical picture. It was marked by the coexistence of delusional beliefs, which increased its complexity and posed many challenges, from both the diagnostic and management aspects.

## Case presentation

Our patient is a 29-year-old, single, unemployed, Indian man. In 2024, he presented to the emergency department via ambulance, which his parents had called after he experienced an episode of loss of consciousness (LOC) at home. A quick medical review of his vital signs and random blood sugar revealed severe hypoglycemia, which was the probable cause of the LOC episode (Table 1).

**Table 1:** Initial vital signs and random blood sugar

Test	Value	Reference range
Temperature	36.5 °C (Tympanic)	-
Heart rate	50 beats per minute	-
Respiratory rate	18/min	-
Blood pressure	102/69 mmHg	-
Oxygen saturation	95%	-
Height	181 cm	-
Weight	46 kg	-
Body mass index	13.7 kg/m²	-
Random blood sugar	3.3 mmol/L	3.9-11.1 mmol/L

Upon taking further history, it became evident that the patient had been experiencing severe obsessions related to feeding (that his gastrointestinal tract had to be fully void of any food or substances, including medications, or he would suffer; however, he did consume water but very conservatively) and contamination before he could have another meal. The patient only agreed to consume a few spoonfuls of home-cooked meals once every few days. These obsessive thoughts led to high levels of anxiety; his solution was to completely refuse to ingest anything, which relieved some of the tension. Following any oral intake, he would spend hours in the bathroom trying to force bowel movements. He denied any use of laxatives or purgative agents to facilitate this.

In addition to the symptoms related to feeding and bowel movements, he would take prolonged showers, lasting up to two hours, multiple times a day, especially after voiding or whenever anything touched his skin. He would also spend hours repetitively washing his hands.

His presentation had been progressively worsening over the past year. His obsessions began appearing more psychotic in nature and were affecting both his physical health and socio-occupational functioning. In addition, he exhibited Schneiderian first-rank symptoms in the form of a delusional mood and delusions of being controlled. He exhibited delusions of control at the time of presentation to the emergency department, possibly beginning earlier when he was not on treatment. The basis of his delusion was that another entity, unknown to him, which was not himself, is what inflicted the cycle of his OCD on him; this entity controlled his thoughts, as these thoughts did not belong to him, and so he could not reverse the experience by himself. Of note, the patient had a previous diagnosis of OCD, but did not provide any medical reports that described his condition. For that reason, we could not relate the current symptomatology to his previous presentation. His presentation was also associated with low mood, poor self-care, and death wishes, with possible suicidal ideation.

Premorbidly, the patient was high functioning and had successfully completed a master’s degree in civil engineering. This episode was possibly precipitated by the social stress of unemployment, as he never found a job after graduating over a year ago, and was perpetuated by poor adherence to medication, limited insight, and a family with high expressed emotion. He was previously physically healthy and denied any use of alcohol or illicit substances. He also did not exhibit any signs of eating disorders or personality disorders prior to the illness. There was no family history of mental illness. The patient lived with his parents, maintained a good relationship with them, though they were noted to be permissive in regards to his medication compliance and seeking medical care.

The patient first experienced symptoms in 2010 while in the tenth grade. Obsessions related to cleanliness, which caused him to wash excessively to reduce his stress, and sexual content, which caused him to feel guilt and shame. This led to a diagnosis of OCD, for which he was medicated. He received outpatient treatment in India, but his adherence to follow-up appointments and medication was inconsistent. His illness has followed a remitting and relapsing course, especially during stressful periods. Notably, there was no past history of aggression, agitation, psychotic or mood symptoms, suicidal ideation, or self-harm behaviors.

On examination, his appearance was that of an emaciated young man with periorbital edema, pedal edema, abdominal distension, and poor grooming. His mental state examination revealed a slow rate of speech with low tone and volume, a low mood with congruent dysphoric affect, and a thought process marked by thought blocking. His thought content was as described above. He did not report any perceptual abnormalities. The patient had impaired reality testing as his obsessions reached a delusional level. He expressed death wishes with possible suicidal ideation and showed poor insight into his condition.

The patient’s body mass index (BMI) at presentation was 13.7 kg/m² (weight: 46 kg, height: 181 cm), and his Yale-Brown Obsessive Compulsive Scale score was 37, corresponding to extreme symptoms of OCD (Table 2).

**Table 2:** Yale-Brown Obsessive Compulsive Scale (Y-BOCS) score

Time Occupied by Obsessive Thoughts	Greater than 8 hrs/day or nearly constant occurrence
Interference Due to Obsessive Thoughts	Incapacitating
Distress Associated With Obsessive Thoughts	Near-constant and disabling distress
Resistance Against Obsessions	Completely and willingly yield to all obsessions
Degree of Control Over Obsessive Thoughts	Obsessions are completely involuntary, rarely able to even momentarily alter obsessive thinking
Time Spent Performing Compulsive Behaviors	More than 3 and up to 8 hrs/day, or very frequent performance of compulsive behaviors
Interference Due to Compulsive Behaviors	Incapacitating
Distress Associated With Compulsive Behavior	Incapacitating anxiety from any intervention aimed at modifying activity
Resistance Against Compulsions	Yield to almost all compulsions without attempting to control them, but with some reluctance
Degree of Control Over Compulsive Behavior	Very strong drive to perform behavior, must be carried to completion, can only delay with difficulty
Total Score: 37	

**Figure 1:**
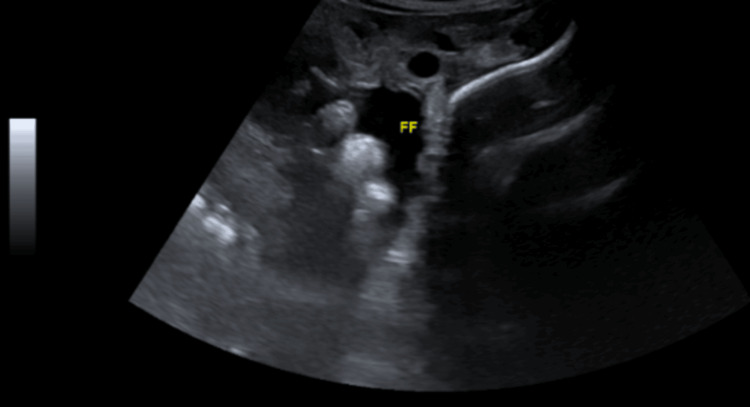
Abdominal ultrasound showing free turbid peritoneal fluid

**Figure 2:**
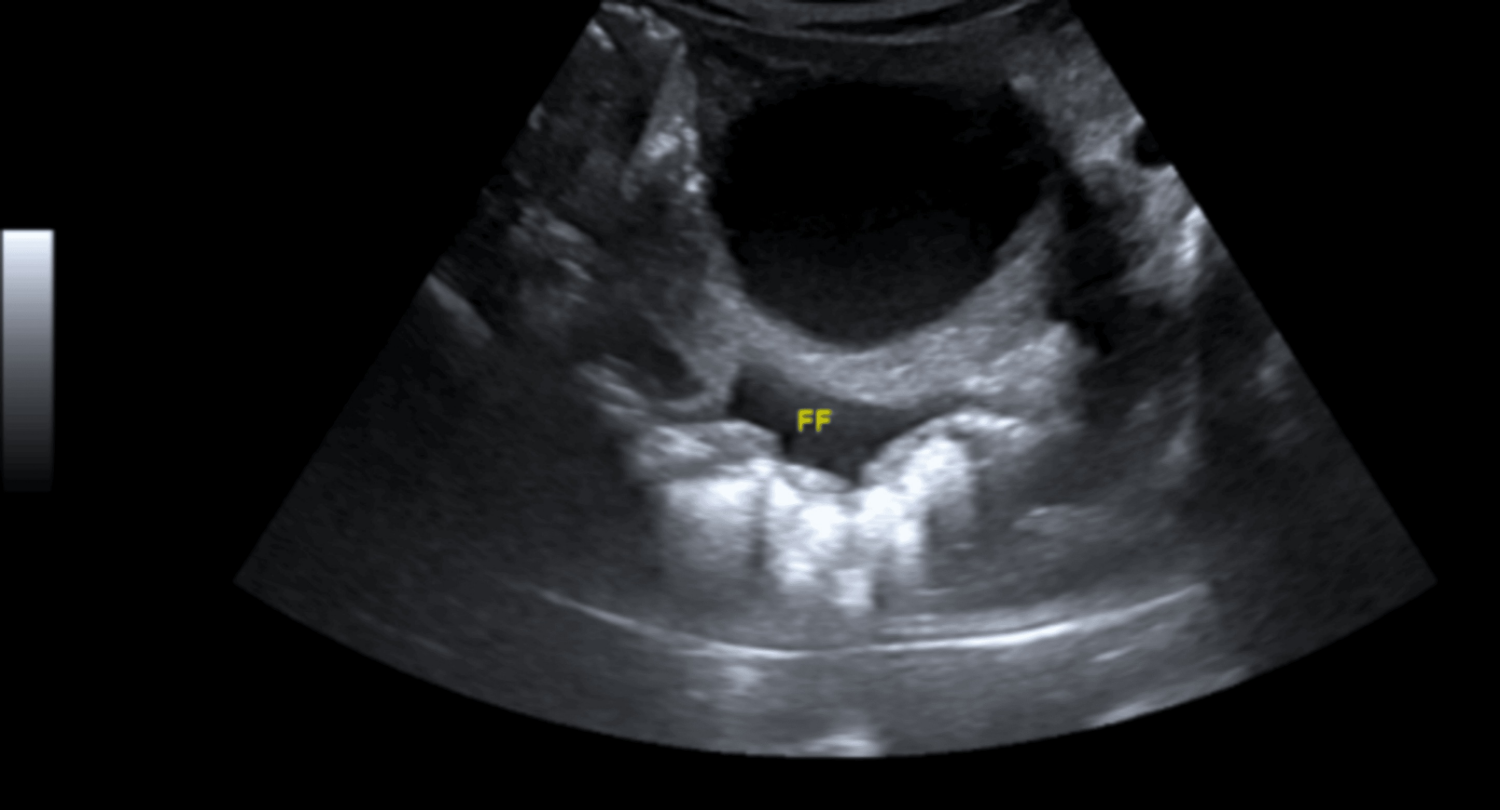
Another view of abdominal ultrasound showing free turbid peritoneal fluid

**Figure 3:**
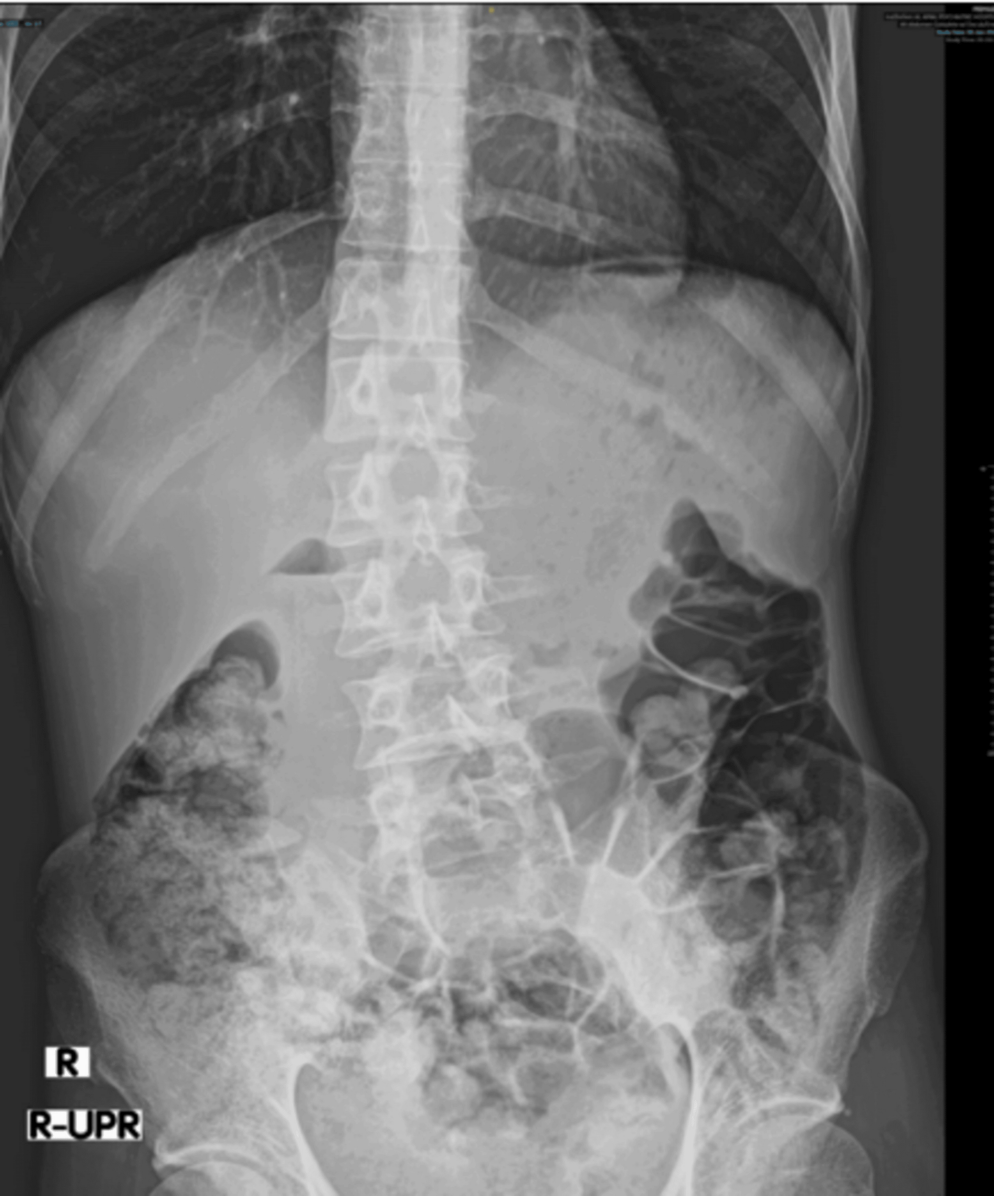
Abdominal X-ray revealing gaseous and fecal matter loading of colonic loops

He was admitted to the general hospital, where he had presented, and was followed up by the medical team and the liaison psychiatry team. Medically, he was managed conservatively. Investigations revealed anemia and hypoproteinemia (Table 3), which were treated with two units of packed red blood cells and albumin infusions. Radiological findings revealed ascites (Figures 1, 2) and fecal loading in the colon (Figure 3). His abdominal findings were not symptomatic. The medical team advised to keep the patient NPO and added a Fleet® enema. He was initiated on a total intake of 2 liters (1 L total parenteral nutrition + 500 mL dextrose saline), less than 1000 K calories per day to avoid refeeding syndrome, as well as 2 g of magnesium, 20 millimoles of phosphate, and albumin-human 20% injection, 100 mL IV daily.

**Table 3:** Initial blood investigations

Test	Value	Reference range
White blood cells	6.33x10(3)/uL	4-10x10(3)/uL
Red blood cells	3.4x10(6)/uL	4.5-5.5x10(6)/uL
Hemoglobin	10.3 g/dL	13-17 g/dL
Hematocrit	29.1%	40-50%
Mean corpuscular volume (MCV)	86 fL	80-100 fL
Mean corpuscular hemoglobin (MCH)	30.4 pg	27-32 pg
Mean corpuscular hemoglobin concentration (MCHC)	35.4 g/dL	31.5-34.5 g/dL
Red cell distribution width (RDW)	15%	11.5-14.5%
Platelet	207x10(3) mcg	150-450x10(3) mcg
Sodium level	135 mmol/L	135-145 mmol/L
Potassium level	4.17 mmol/L	3.5-5.1 mmol/L
Chloride level	100 mmol/L	98-107 mmol/L
Blood urea nitrogen (BUN)	16.4 mmol/L	2.5-6.4 mmol/L
Creatinine	37 umol/L	80-115 umol/L
Uric acid	347 umol/L	208-428 umol/L
Glucose	3.9 mmol/L	3.9-11.1 mmol/L
Estimated glomerular filtration time (eGFR)	149	
Total protein	56 g/L	64-882 g/L
Albumin level	28 g/L	34-50 g/L
Bilirubin total	19.7 umol/L	3-17 umol/L
Alanine transaminase (ALT)	186 IU/L	16-63 IU/L
Aspartate aminotransferase (AST)	196 U/L	15-37 U/L
Alkaline phosphatase	51.75 IU/L	46-116 IU/L
Cortisol AM	671.41 nmol/L	145.4-619.4 nmol/L
Adrenocorticotropic hormone (ACTH)	<1.11 pmol/L	0.0-10.0 pmol/L
T4 free	18.4 pmol/L	9-20 pmol/L
T3 free	4.72 pmol/L	3.5-6.5 pmol/L
Thyroid-stimulating hormone (TSH)	0.92 mIU/L	0.55-4.78 mIU/L

Due to the nature of his presentation, he was initiated on olanzapine at a dose of 5 mg daily, then tapered up to reach 5 mg twice daily, which was given to tackle his psychotic symptoms as described above, and to help increase his appetite and oral intake. The patient began to accept oral feeds, but due to inadequate monitoring of his nutritional status, he developed refeeding syndrome, which, in his case, comprised hypokalemia, hypochloremia, hypomagnesemia, and hypophosphatemia (Table 4). He was kept nil by mouth for one day until his electrolytes were corrected. In addition to the abovementioned fluids and electrolytes, he was also started on potassium chloride 40 mmol IV daily, vitamin B1 parenteral 100 mg IV daily, and folic acid supplementation. After his electrolytes were corrected, oral feeding was reintroduced under the supervision of the nutrition department.

**Table 4:** Bloodwork indicating refeeding syndrome

Test	Value	Reference range
Sodium level	141 mmol/L	135-145 mmol/L
Potassium level	2.72 mmol/L	3.5-5.1 mmol/L
Chloride level	22.88 mmol/L	98-107 mmol/L
Magnesium level	0.73 mmol/L	0.74-0.99 mmol/L
Phosphorus level	0.64 mmol/L	0.84-1.52 mmol/L
Calcium level	1.99 mmol/L	2.12-2.52 mmol/L

The patient’s family expressed the wish to take him to their home country, as the patient and his parents were expatriates for many years in the country of residence, for continued inpatient treatment, and they signed him out of the hospital against medical advice on the day of their flight. Unfortunately, he was brought back by his parents a couple of weeks later with the same presentation, as he had not wanted to travel, and they had not insisted. Additionally, while at home, he did not adhere to his medications due to obsessions related to feeding, which extended to his medications as well.

The patient was then admitted to a psychiatric facility after receiving medical clearance, where he stayed as an inpatient for 18 days. He was started on fluvoxamine alongside olanzapine to adhere to the OCD treatment guidelines, reaching doses of 100 mg twice daily and 10 mg at bedtime, respectively, and stayed on them while inpatient.

At the time of discharge, he continued to experience excessive delays in completing tasks due to obsessive-compulsive tendencies with persistent thoughts of contamination and compulsions to shower frequently. Fear of contamination was the only persistent thought. He noted a marked improvement in terms of mood, his suicidal ideas subsided, and his judgment improved, as he was compliant with his medications and willing to take them.

## Discussion

Studies have historically found a correlation or continuum between eating disorders and OCD. A study published in 2009 found that 11.4% of patients diagnosed with OCD had a comorbid eating disorder [6]. Atypical presentations involving both OCD and eating disorders coexisting have been reported in the literature. A case report published in 2009 described an atypical form of OCD that manifested with symptoms resembling anorexia nervosa, as the obsessions were all related to healthy dietary habits and food contamination. Over time, the patient’s obsession with contamination and aversion to eating intensified and expanded to include avoidance of physical contact with various objects [7].

Another paper reported a case of a middle-aged woman who suffered from treatment-refractory anorexia nervosa and OCD. Her obsessions included preoccupations with food, weight, and body shape; she also had a distorted body image and absence of menstruation. Her compulsions involved restrictive eating, laxative use, and excessive exercise [8].

A summary of the anorexia nervosa diagnostic criteria provided in the Diagnostic and Statistical Manual of Mental Disorders, Fifth Edition, Text Revision (DSM-5-TR) involves restriction of energy intake, leading to significantly low body weight for age, sex, and health context, an intense fear of gaining weight or behaviors preventing weight gain despite being underweight, and a distorted body image, overemphasis on weight in self-evaluation, or denial of the severity of low body weight [3]. What helped eliminate the diagnosis in our patient was that he did not meet the diagnostic criteria: he experienced no distortion in body image, had no fear of weight gain, and did not have episodes of bingeing or purging.

Moreover, there is a diagnostic challenge in differentiating between obsessions with poor insight and delusions. A delusion is defined as a false belief based on incorrect inference about external reality, firmly held despite clear contradictory evidence, and not explained by cultural or religious norms. An obsession is defined as a recurrent and persistent thought, urge, or image that is experienced as intrusive and unwanted, causing marked anxiety or distress, and which the individual attempts to ignore or suppress.

Delusions are generally easier to identify, while descriptions of obsessions are often broad and vague, and there is considerable clinical overlap between the two [9]. A meta-analysis published in 2013 estimated that 13.6% of patients diagnosed with schizophrenia also had a comorbid OCD diagnosis, and 30.7% had comorbid obsessive-compulsive symptoms [10]. A nationwide study conducted in Denmark and published in 2014 found that 2.75% of individuals diagnosed with schizophrenia had a prior hospital contact for OCD [11].

As per the schizophrenia diagnostic criteria provided in the Diagnostic and Statistical Manual of Mental Disorders, Fifth Edition, Text Revision (DSM-5-TR), the diagnosis is made when two or more of the following are present for at least one month: delusions, hallucinations, disorganized speech, grossly disorganized or catatonic behavior, and negative symptoms. At least one symptom must be delusions, hallucinations, or disorganized speech. There must be significant social or occupational dysfunction, with continuous signs of disturbance persisting for at least six months. Furthermore, delusional disorder is also characterized by the presence of one or more delusions lasting at least one month. Functioning is not markedly impaired, and behavior is not bizarre or odd outside the delusional context [3]. Our patient did not fulfill the remaining diagnostic criteria for either at the time of the encounter, but schizophrenia should still be considered as a differential diagnosis, particularly in the future, depending on the progression of the case.

Management of OCD should generally be approached using a biopsychosocial model. According to the American Psychiatric Association (APA), the first line of treatment involves either cognitive-behavioral therapy, mainly exposure and response prevention (ERP), or selective serotonin reuptake inhibitors (SSRIs), or a combination of both. The second line of treatment, in cases of moderate, little, or no response, includes adding further psychological interventions, switching to another antidepressant agent (i.e., another SSRI, mirtazapine, venlafaxine, etc.), or augmenting with a second-generation antipsychotic. If both first- and second-line treatments fail, transcranial magnetic stimulation, deep brain stimulation, and finally neurosurgery may be considered, all of which have been reported in the literature and shown to be effective [8,12].

In our patient’s case, the treatment approach had to be undertaken with great caution due to the risk of medical complications associated with malnutrition. The primary concern was refeeding syndrome, which he experienced within his first week of hospitalization. Refeeding syndrome is a complication that results from fluid and electrolyte shifts during aggressive nutritional rehabilitation. It is caused by nutritional replenishment following periods of starvation and is marked by a rapid increase in insulin levels, leading to electrolyte imbalances -- hypokalemia, hypophosphatemia, hypomagnesemia -- and decreased levels of thiamine. Each of these can cause complications such as cardiac arrhythmias, fatigue, respiratory depression, Wernicke’s and Korsakoff syndromes, paresthesia, or convulsions [13].

This case report highlights a unique strength, as it presents findings not previously reported. A notable limitation, however, was the absence of psychological treatment during the hospital stay. We recommend that clinicians managing similar cases closely monitor weight and BMI, alongside the use of psychometric tools to track progress. A comprehensive bio-psycho-social model should be applied for optimal treatment outcomes, with a multidisciplinary approach, including an internist and a nutritionist, to manage malnutrition safely under close observation. Finally, psychoeducation should be offered to the patient and their family members.

## Conclusions

In conclusion, this case highlights a complex and atypical presentation of OCD, where obsessive thoughts about food intake and contamination led to severe malnutrition. The patient’s presentation posed unique challenges in both diagnosis and treatment, particularly as his condition was perpetuated by poor insight, poor adherence to treatment, and psychosocial stressors. The primary aim of sharing this case was to highlight atypical manifestations of OCD, especially those involving food refusal, which may mimic other disorders, such as eating disorders, leading to misdiagnosis and mismanagement.

Attempting to treat the malnutrition without close monitoring of the patient’s oral intake and electrolytes resulted in the development of refeeding syndrome. This, in turn, further complicated the clinical course and prolonged his stay in a general hospital for medical care. This reinforces the importance of close medical and nutritional monitoring during the reintroduction of oral feeding in patients presenting with malnourishment due to extended periods of poor intake. In such cases, it is also advisable to implement a multidisciplinary approach to create an optimal management plan. Ultimately, this case demonstrates the critical need for integrated psychiatric and medical care, ongoing psychoeducation, and proactive family involvement to improve both short- and long-term outcomes for patients.
